# Standardizing Health Workforce Data in Canada: Legal and Regulatory Levers for Harmonized Collection and Sharing

**DOI:** 10.1177/08404704251403158

**Published:** 2026-02-02

**Authors:** Alexandra Lyn, Kathleen Leslie, Arthur Sweetman, Geetanjali Sharma, Sarah Lazin, Gwenneth Feeny, Ivy Lynn Bourgeault

**Affiliations:** 1 School of Business, 3151 MacEwan University, Edmonton, Alberta, Canada; 2 Faculty of Health Disciplines, 1349 Athabasca University, Athabasca, Alberta, Canada; 3 Department of Economics, 3710 McMaster University, Hamilton, Ontario, Canada; 4 Faculty of Law, 56005 University of Ottawa, Ottawa, Ontario, Canada; 5 3710 McMaster University, Hamilton, Ontario, Canada; 6 School of Sociological and Anthropological Studies, 151181 University of Ottawa, Ottawa, Ontario, Canada

## Abstract

There is a growing awareness of the benefits of comprehensive, standardized, and accessible data on the health workforce to support more timely and robust planning. We found that provincial regulation and data privacy legislation could be better aligned to strengthen the infrastructure for high-quality health workforce planning data. This article identifies existing legal and regulatory mechanisms that enable the collection and sharing of more standardized health workforce data. We propose a framework that enables the collection and sharing of standardized data by scaling up existing leading practices in certain provinces into a more cohesive approach. Key facilitators include umbrella legislation, privacy frameworks that contemplate data use for workforce planning, efforts to collect anti-discrimination data, and secure data access infrastructure. Together, these facilitators support a viable foundation for improved health workforce data standardization and utilization for planning to improve healthcare delivery across Canada in the existing legal context.

## Introduction

There is a growing understanding, in Canada and globally, of the need for more accurate, comprehensive, standardized, and accessible data on the health workforce to support more timely, responsive, and proactive health workforce planning.^1^ Effective health workforce planning is evidence informed, needs-based, multi-professional, locally relevant, iterative, and interactive, and it utilizes multiple sources of evidence to ensure it is robust.^2^ Better planning requires high-quality, standardized, and timely data.

Notwithstanding the central role of health workers in delivering effective healthcare, Canada’s health workforce data collection, infrastructure, and analytics lag behind comparable Organisation for Economic Co-operation and Development (OECD) countries.^3^ The federated nature of Canada’s healthcare system (including health practitioner regulatory systems) contributes to a siloed, unstandardized approach to health workforce data.^4^ This results in the fragmented and uneven collection of registry data for health workers across Canada, with incompatible datasets^5^ that make robust health workforce planning challenging, and sometimes infeasible. Further, reconciling data sources across jurisdictions, when feasible, limits data quality and timely accessibility. A consequence of these data limitations is that health workforce planning in Canada tends to be siloed by profession and/or jurisdiction resulting in less effective decision-making. A coordinated health workforce data enhancement initiative is needed to ensure that high quality, standardized data across professions and jurisdictions is available to improve workforce planning and healthcare for everyone in Canada.

Beyond collection, health workforce data needs to be accessible to a range of health planners in government, communities, and academia. The complexity of Canada’s privacy laws, however, can hamper the accessibility of workforce planning data. Much like Canada’s regulation of health practitioners, the Canadian privacy landscape is composed of a patchwork of federal, provincial, and territorial laws.^6^ This variability adds complexity and appreciable transactions costs in accessing health workforce data.

The purpose of this article is to identify existing legal and regulatory mechanisms that enable the collection and sharing of health workforce data for planning. We draw upon health practitioner regulation and data privacy legislation from six provinces that represent distinct approaches to data collection and sharing.

## Methodological Approach

We undertook a comparative environmental scan of the existing grey literature and legal frameworks in the provinces of British Columbia, Alberta, Saskatchewan, Ontario, Prince Edward Island, and Nova Scotia. These represent geographic and population differences, and different privacy and regulatory schemes. The federated nature of Canada’s health system necessitated that the legal analysis be conducted at the sub-national level. To ensure the scope of this analysis remained feasible, we analyzed a subset of provinces with umbrella regulatory legislation (defined below) of various maturities as well as ones that were undergoing reform. The legal frameworks of the Canadian territories are beyond the scope of this discussion. Initial searches were completed between October 2022 and October 2023 and updated in September 2025.

We first analyzed professional regulatory schemes across the case study provinces to identify what data regulatory bodies are required to collect and share, which may be distinct from what regulators undertake of their own accord. We then analyzed privacy and data protection legislation in these case study provinces to consider the interaction with health practitioner regulators’ data collection requirements. We conducted an iterative legal analysis relevant to professional regulation, data collection, and privacy. We consulted secondary authorities and accessed case law and statutes using Westlaw and CanLII.

## Findings

Provincial regulatory authorities are required by provincial legislation to collect personal information about their respective health workforce through their registries, as described in Table 1. Provinces differ not only in the legislative mechanisms used to regulate health practitioners but also in the set of practitioners that are regulated, the nature of data collected for these registries, and the circumstances under which disclosure is specifically contemplated.^5^ Notwithstanding this variability, the data already being collected by provincial regulators could serve as a helpful and cost effective starting point for making more robust data‐driven workforce planning decisions if the existing information could be enhanced and accessed, with appropriate privacy and security safeguards, and better utilized by qualified analysts, researchers, and policy-makers.

**Table 1.** Province-Level Legislation for Regulating Health Practitioners and Data Privacy

Jurisdiction	Legislation regulating health professionals	Privacy legislation	Applicable sector
Canada	--	*Personal Information Protection and Electronic Documents Act,* SC 2000, c 5 (PIPEDA)	Private
		*Privacy Act,* RSC 1985, c P-21	Public
British Columbia	*Health Professions Act,* RSBC 1996, c 183	*Freedom of Information and Protection of Privacy Act,* RSBC 1996, c 165	Public
		*Personal Information Protection Act,* SBC 2003, c 63	Private (substantially similar to PIPEDA)
		*E-Health (Personal Health Information Access and Protection of Privacy) Act,* SBC 2008, c 38	Health
		*Privacy Act,* RSBC 1996, c 373	Private (tort)
Alberta	*Health Professions Act,* RSA 2000, c H-7	*Protection of Privacy Act,* SA 2024, c P-28.5	Public
		*Access to Information Act,* SA 2024, c A-1.4	
		*Personal Information Protection Act,* SA 2003, c P-6.5	Private sector (substantially similar to PIPEDA)
		*Health Information Act,* RSA 2000, c H-5	Health Records
		*Protecting Victims of Non-consensual Distribution of Intimate Images Act,* SA 2017, c P-26.9	Private
Saskatchewan	*The Regulated Health Professions Act*. Vol 2025, c 12	*The Freedom of Information and Protection of Privacy Act,* SS 1990-91, c F-22.01	Public
		*Local Authority Freedom of Information and Protection of Privacy Regulations,* RRS c L-27.1 Reg 1	Public (Municipal)
		*The Health Information Protection Act,* SS 1999, c H-0.021	Health
		*The Privacy Act,* RSS 1978, c P-24	Private (tort)
Ontario	*Regulated Health Professions Act,* SO 1991, c 18	*Freedom of Information and Protection of Privacy Act,* RSO 1990, c F.31	Public
	For Personal Support Workers: *Health and Supportive Care Providers Oversight Authority Act*, 2021, SO 2021, c 27, Sch 2	*Municipal Freedom of Information and Protection of Privacy Act,* RSO 1990, c M.56	Public (Municipal)
		*Personal Health Information Protection Act,* 2004, SO 2004, c 3, Sch A	Health
	For Social Workers and Social Service Workers: *Social Work and Social Service Work Act,* 1998, S.O. 1998, c. 31	*Part X, Child, Youth and Family Services Act,* 2017, SO 2017, c 14	Child and Youth Sector
Prince Edward Island	*Regulated Health Professions Act,* RSPEI 1988, c R-10.1	*Freedom of Information and Protection of Privacy Act,* RSPEI 1988, c F-15.01	Public
		*Health Information Act,* RSPEI, 1988, c H-1.41	Health
Nova Scotia	*Regulated Health Professions Network Act,* SNS 2012, c 48 and profession specific statutes	*Freedom of Information and Protection of Privacy Act,* SNS 1993, c 5,	Public
		*Personal Information International Disclosure Protection Act,* SNS 2006, c 3	Public
		*Personal Health Information Act,* SNS 2010, c 41	Health
		*Municipal Government Act,* SNS 1998, c 18, Part XX	Public (Municipal)

Table Reproduced, in part, from: Power, Michael, The Law of Privacy, 3rd Edition, (Toronto LexisNexis Canada Inc, 2021) at 7-9

**Figure 1.**
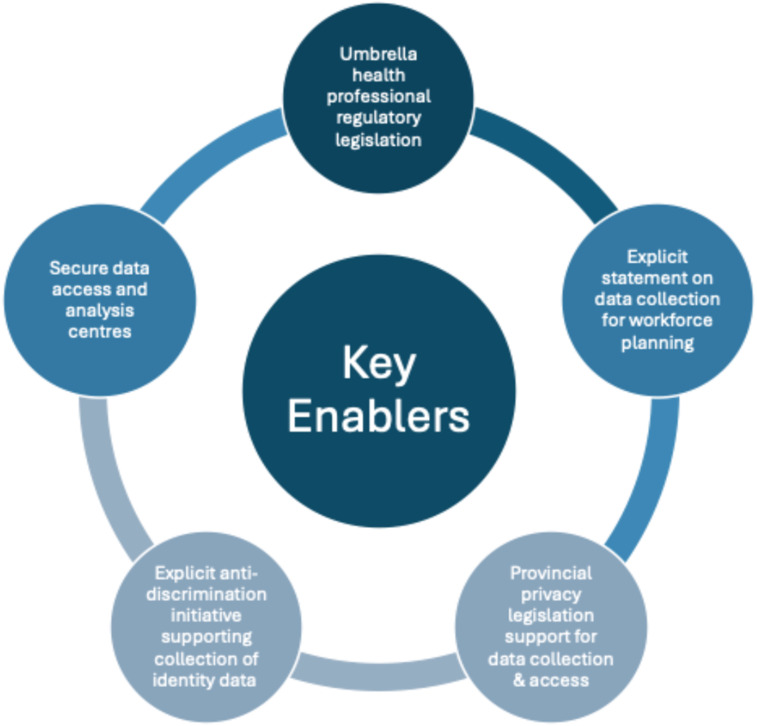
A Framework of Enablers of the Collection of More Comprehensive, Standardized, and Accessible Health Workforce Data for Planning

Health practitioner regulation falls under provincial/territorial authority. Therefore, depending on their current regulatory and data collection frameworks, some of these jurisdictions may require legislative changes to standardize and improve data quality. Many jurisdictions could achieve this with minor legal amendments rather than major overhauls. Figure 1 summarizes the key enablers that together form a framework for improved data collection and accessibility.

## Enabler 1: Umbrella Legislation

“Umbrella legislation” refers to a single overarching statute applying to all regulated health professions with regulations for each individual profession promulgated thereunder. Using umbrella legislation to harmonize the regulation of health practitioners across each province or territory can support the implementation of comprehensive and standardized health workforce data collection. Umbrella legislation provides a “template approach”^7^ offering greater uniformity amongst regulated professions and provides a more efficient means of updating health legal frameworks because governments can amend the overarching statute or enact a single regulation applicable to all regulated professions.

Umbrella legislation is already used in many Canadian jurisdictions. For example, in Ontario, the *Regulated Health Professions Act, 1991*^8^ covers 26 regulatory authorities that regulate 31 professions.^9^ Each of these 26 regulators also has profession-specific acts that define the scope of practice and protected titles for the professions and establishes the mandate of the regulators. A 2008 amendment to Ontario’s *Regulated Health Professions Act* explicitly allows for the collection of standardized information for the purpose of health workforce planning by the government.^8^

Alberta also has an umbrella legislative framework—the *Health Professions Act, 2000*^10^ (“*HPA*”) and the *Disclosure of Information Regulation*^11^—that lends itself well to the implementation of the collection of more comprehensive and standardized health workforce data without the need for a complete legislative overhaul. Alberta uses the *HPA* to regulate 29 health professions and collects registrant data from each profession under section 33 of the *HPA* and under the Schedule to the Regulation.

British Columbia recently enacted the *Health Professions and Occupations Act* (*HPOA*),^12^ which will come into force on April 1, 2026. As part of the regulatory modernization efforts with the *HPOA*, BC’s 20 health practitioner regulators have been (or are in the process of being) amalgamated into 6.^13^

Finally, Saskatchewan recently transitioned its regulatory approach with the enactment of *The Regulated Health Professions Act*, *2025*,^14^ a piece of umbrella legislation aimed at consolidating the existing “21 individual Acts regulating 28 health professions governed by 26 regulatory bodies” in the province of Saskatchewan.^15^ In doing so, Saskatchewan acknowledged that the new umbrella framework brings Saskatchewan, “in alignment with what is considered best practice, similar to legislation already in place in other provinces in Canada.”^15^ Notably, the *Regulated Health Professions Act* does not prescribe nor harmonize the information to be collected by regulators. Instead, it maintains that regulators may enact bylaws requiring members to provide certain information and delineate how that information can be used and disclosed.^14^ The *Act* also empowers regulators to make bylaws governing the compiling and sharing of de-identified information.^14^

In provinces with umbrella legislation for health practitioner regulation, expanded health workforce data collection could be legislated via an amendment to the umbrella legislation or regulation, as was undertaken in 2008 in Ontario.

## Enabler 2: Health Workforce Planning Explicitly Contemplated for Data Use and Disclosure in Provincial Health Regulatory Frameworks

Four case study provinces (Ontario, British Columbia, Alberta, and Nova Scotia) specifically contemplate the collection, use, and disclosure of registrant data for workforce planning purposes in their provincial legislation regulating health professionals. The 2008 amendment to Ontario’s legislation created a minimum dataset for health practitioners in Ontario called the Health Professions Database. This database has 59 standard data elements that all colleges, with an exception for medicine, are required to collect, including certain demographic information, geography, education, and employment.

Alberta’s *HPA* permits registrant information to be used for the purposes of “planning and resource allocation, health system management, public health surveillance and health policy development, or providing information about regulated health professionals to the public” (section 122).^16^ British Columbia’s statutes^12,17^ allow for the sharing of information with the Minister of Health—with the exception of personal information (defined as recorded information about an identifiable individual other than contact information)^12,17^—for the purposes of health workforce management.^12,17^ Similarly, Nova Scotia requires the Nova Scotia Regulated Health Professions Network (composed of bodies corporate to govern regulated health professions in Nova Scotia) to “provide information to other bodies that will facilitate improvements in healthcare delivery or regulation… and encourage voluntary collaboration among Network members for any purpose that serves the public interest”^18^ although the statute does not specify which information should be disclosed, nor does it identify activities that serve the public.

More broadly, the collection of existing “administrative data”—that is, “data that are already in existence, having been collected for other purposes”—by government institutions is of growing interest nationally but raises significant legal and public policy considerations.^19^ Although much Canadian healthcare data exists within the public sector, a national framework for public sector use of private sector data would be beneficial, “perhaps in the form of a ministerial directive or formal guidance” to facilitate pan national workforce planning that leverages existing data.^19^

## Enabler 3: Compliance with Privacy Laws Possible through Data Anonymization and Aggregation

The collection, use, and disclosure of registrant data would need to comply with both federal and provincial privacy statutes, as summarized in Table 1.^20^ Notably, compliance with privacy laws can be achieved in all the provinces analyzed if the data are sufficiently anonymized such that the dataset cannot be linked back to an identifiable individual. This is because one of the thresholds for privacy protections in Canada is that the data relate to an “identifiable individual,”^21‐24^ that is, an individual where there is a serious possibility of identification from the data itself or in combination with other information.^25^

At first blush, the anonymization of registrant data may appear to be a simple and streamlined solution, but it is far from a panacea given the complexities of Canada’s privacy regime and the data needs of high-quality planning. The concept of anonymization exists on a spectrum, and the risk of re-identification after the data has been anonymized is not static, especially considering rapid technological advancements. Furthermore, the utility of the data for workforce planning purposes is commonly compromised if the data are too generic to support meaningful analysis. This means that much useful data analysis using so-called micro- or individual-level data, even when using data without direct individual identifiers, holds the possibility of “residual disclosure” (i.e., individual identification in data without individual identifiers by using ancillary information). Such analysis, therefore, needs to occur in a secure environment (see enabler 5). The results of such secure analyses are reasonably straightforwardly made anonymous/aggregated prior to being made public (commonly referred to as vetting or disclosure). Beyond this type of analysis, Alberta’s provincial privacy legislation offers a potential solution to the perils of anonymized data by specifically authorizing, “the collection, use, and disclosure of personal information without consent if [it is] authorized or required under an information code of a professional regulatory organization,” thus removing the need for anonymization.

## Enabler 4: Data Collection in Furtherance of Anti-Discrimination Initiatives

There is a growing demand on professional regulators to adhere to anti-discrimination standards to help assess and address systemic barriers to equitable access, inclusion, and fair treatment.^26^ Health workforce information on gender, race/ethnicity, disability, and other intersecting employment equity identifiers can be collected to meet such standards; health workforce regulators can thereby design bias-awareness policies, promote fair registration requirements, and monitor outcomes.^15,16^ Table 2 summarizes the key facilitators in select provinces that support the inclusive collection and sharing of standardized data for health workforce planning.

**Table 2.** Summary of Key Facilitators in Case Study Provinces

Province	Enabler				
	Legislation regulating health professionals		Anti-racism legislation supporting collection of identity data?	Secure data access centres?	Privacy legislation
	Umbrella legislation?	Explicit statement on data collection for workforce planning?			Compliance through sufficiently anonymized data possible?
Ontario	Yes^a^	Yes^b^	Yes^c^	Yes^d^	Yes
British Columbia	Yes^e^	Yes^f^	Yes^g^	Yes^h^	Yes
Alberta	Yes^i^	Yes^j^	No	Yes^k^	Yes
Saskatchewan	Yes^l^	No^m^	No	Yes^n^	Yes
Nova Scotia	Partially^o^	Yes^p^	Yes^q^	Yes^r^	Yes
PEI	Yes^s^	No	No	Yes^t^	Yes

^a^*Regulated Health Professions Act,* SO 1991, c 18. Note that personal support workers and social workers are regulated separately under the *Health and Supportive Care Providers Oversight Authority Act*, 2021, SO 2021, c 27, Sch 2 and *Social Work and Social Service Work Act,* 1998, S.O. 1998, c. 31 respectively.

^b^*Regulated Health Professions Act*, 1991, SO 1991, c 18, s 36.1(1).

^c^See *Anti-Racism Act, 2017, SO 2017, c 15,* https://canlii.ca/t/569mp *retrieved on 2025-09-08;* Data Standards for the Identification and Monitoring of Systemic Racism, Anti-Racism Directorate, Ontario, https://www.ontario.ca/document/data-standards-identification-and-monitoring-systemic-racism

^d^Institute for Clinical Evaluation Sciences (ICES) Data Centre, https://www.ices.on.ca/services-for-researchers/

^e^*Health Professions Act,* RSBC 1996, c 183.

^f^*Health Professions and Occupations Act*, Bill 36-2022, ss 491 (1) and (2), https://www.bclaws.gov.bc.ca/civix/document/id/bills/billsprevious/3rd42nd:gov36-3; *E-Health (Personal Health Information Access and Protection of Privacy) Act*, SBC 2008, c 38, https://canlii.ca/t/5571s retrieved on 2025-09-08 also allows for the collection and use of personal health information for the planning, maintenance, and/or improvement of health services (see s,4(g)).

^g^*Anti-Racism Data Act,* SBC 2022, c 18.

^h^Population Data BC, https://www.popdata.bc.ca/

^i^*Health Professions Act,* RSA 2000, c H-7.

^j^*Health Professions Act,* RSA 2000, c H-7 s 122.

^k^Connect Care, Alberta Health Services, https://www.albertahealthservices.ca/cis/cis.aspx; see also for more illustrations at Table 2, Sandhu, N., Whittle, S., Southern, D. A., Li, B., Youngson, E., Bakal, J. A. & Eastwood, C. A. (2023). Health data governance for research use in Alberta. *International Journal of Population Data Science*, *8*(4), 2160.

^l^*The Regulated Health Professions Act*, SS 2025, c 12.

^m^*The Provincial Health Authority Act,* SS 2017, c P-30.3, s 7(6).

^n^Health Research Data Platform–Saskatchewan (HDRP-SK), https://www.scpor.ca/hrdpsk

^o^*Regulated Health Professions Network Act,* SNS 2012, c 48 and profession specific statutes.

^p^*Regulated Health Professions Network Act*, SNS 2012, c 48, s 6(f) and 6(g). Note that the statute does not specify which information should be disclosed, nor does it expand upon activities that are considered to serve the public.

^q^*Dismantling Racism and Hate Act*, SNS 2022, c 3, s 10.

^r^Health Data Nova Scotia, https://medicine.dal.ca/departments/department-sites/community-health/research/hdns.html

^s^*Regulated Health Professions Act,* RSPEI 1988, c R-10.1.

^t^Secure Island Data Repository, https://sidrpei.ca/

British Columbia’s anti-racism legislation is a pioneering force for data collection in support of anti-discrimination initiatives, including, but not limited to equity-based health workforce planning initiatives as mentioned above. Specifically, BC’s *Anti-Racism Data Act,*^27^ which became law on June 2, 2022, and BC’s *Anti-Racism Act*^28^ which became law on May 16, 2024. British Columbia’s *Anti-Racism Data Act* permits a public body (being a ministry of the government of British Columbia)^29,30^ to “collect personal information [meaning information about an identifiable individual other than contact information] for the purposes of identifying and eliminating systemic racism and advancing racial equality.”^30^ Collection of an individual’s personal information under this *Act* is voluntary.^30^ Information collected in accordance with the *Anti-Racism Data Act* can be, “[used] for the purposes of identifying and eliminating systemic racism and advancing racial equity [or disclosed] … to another public body or an Indigenous governing entity in order for that personal information to be used for the purposes of identifying and eliminating systemic racism and advancing racial equity.”^30^ The data collected under this *Act* will be used to inform the provincial Anti-Racism Action Plan (as required by section 3 of the *Anti-Racism Act*).^29^

The *Anti-Racism Data Act* expands upon the relatively narrow data permitted to be collected under BC’s HPOA in furtherance of anti-discrimination initiatives. The *HPOA* in BC allows the minister to request statistical and other information (excluding personal information) for the purposes of monitoring discrimination and evaluating anti-discrimination measures. This includes information respecting the demographic composition of regulated health practitioners, applicants to become regulated health practitioners, employees of regulated health practitioners, and patients.^12^

Ontario also has legislation aimed at reducing systemic racism in its public sphere: the *Anti-Racism Act,* which requires the Ontario government to maintain an *Anti-Racism* strategy.^31^ The standard for collection, use, and management of information, including personal information, under this *Act* is set forth by the Data Standards for the Identification and Monitoring of Systemic Racism^32^ as required by section 6 of the *Act.* As part of broader public sector initiatives, under the *Anti-Racism Act*, 2017, some^33^ regulatory colleges in Ontario are undertaking efforts to promote the collection of social variables data (e.g., identity data including race based data) and to monitor, and design interventions that aspire to reduce, systemic racism and discrimination in registration and practice. For regulated health occupations, the anti-racism legislation builds on Ontario’s *Fair Access to Regulated Professions and Compulsory Trades Act, 2006*, and corresponding “fair access” amendments to its *Regulated Health Professions Act*. Ontario’s Office of the Fairness Commissioner played a key implementational role on this front and highlights the connection between data collection and evidence-informed action.^34-36^

These examples of initiatives and legislative efforts aiming to correct inequities facilitate, and illustrate, the collection of inclusive health workforce data and emphasize the value of standardizing and strengthening the infrastructure for data governance and healthcare quality. While these examples of legislative efforts are currently only present in some provinces, pan-Canadian health workforce planning guided by data standards would support other provinces to contribute to anti-discrimination initiatives more broadly.

## Enabler 5: The Availability of Secure Data Access Infrastructure

All case study provinces operate secure data centres with linkages across the federated system facilitated by Health Data Research Network (HDRN) Canada (https://www.hdrn.ca/en/). Federally, there is also Statistics Canada’s Research Data Centre network (https://crdcn.ca/), which collaborates with the Canadian Institute for Health Information (CIHI) and other organizations. Further, some hospitals and other organizations support secure analysis sites (e.g., https://www.hpc4health.ca/).

At present, health workforce databases are not normally stored in these environments^37^ (e.g., Population Data BC; https://www.popdata.bc.ca/), with the notable exception of nursing, social work, and paramedic data housed by DataNB.^38,39^ Secure data access centres could play a critical role in making health workforce databases accessible to planners and researchers to the benefit of all Canadian residents. They can provide access to de-identified and ideally standardized data from multiple sources while preventing residual disclosure and addressing security/privacy that is de-identified and may be longitudinal. Analysis is conducted in secure environments that ensure privacy protection (e.g., PopData in BC).^38,39^ Equity focused governance can also be supported.

Data sharing across sectors, ministries, and regulatory bodies could be facilitated together with promoting standardization—including Minimum Data Standards (MDSs)—for improved interoperability, and cross profession, sector-focused and inter-jurisdictional analysis.

Canada has made progress towards national databases of practitioner regulatory data (e.g., National Registry of Physicians^40^ and NURSYS Canada^41^ https://portal.nursyscanada.ca/). While currently focused on supporting mobility of practitioners and sharing of disciplinary and registration data between regulators, these databases could also support health workforce planning if aggregate or de-identified data became accessible to researchers and policy-makers in future iterations.^42^ These existing databases can also be leveraged to support an MDS framework at a pan-Canadian level.

## Limitations

While instructive to review six provinces and necessary given time and funding constraints, the main limitation of our review was the exclusion of the remaining provinces and territories in Canada. Future work should go further to review practices of individual regulatory authorities, particularly around the collection of demographic data, to understand and compare current data holdings. We also do not discuss next steps for implementation in this article, though this is part of our team’s broader project which has co-developed an enhanced minimum data standard for health workforce planning across Canada.^43^

## Conclusion

While Canada’s federated health practitioner regulatory landscape creates challenges for data harmonization, it also offers numerous entry points for reform. Key legal and institutional facilitators already exist across several provinces, including umbrella legislation, privacy frameworks that contemplate data use for workforce planning, efforts to collect anti-discrimination data, and secure data access infrastructure. Together, this creates a viable foundation for improved health workforce data standardization for better health system planning and ultimately better health for everyone in Canada.

Rather than requiring wholesale legislative overhaul, targeted amendments and coordinated action can significantly advance standardized, equitable, and privacy-conscious health workforce data systems. Model legislation specifying the data to be collected by regulators, and the purposes for which the data should be used and disclosed, could be used to guide this process. Provinces and territories are encouraged to adopt these leading practices to strengthen health workforce data collection, planning, and care delivery across Canada.

Another key facilitator to enhanced health workforce data is the implementation of an MDS that would align the health workforce information being collected across jurisdictions and provider groups in Canada. A revised MDS from CIHI (2022) being enhanced in partnership with ongoing research led by the Canadian Health Workforce Network will be highly augmentative in this regard.^20^ Understanding and comparing the variables currently contained within regulatory authority registration databases to begin harmonization efforts would be an important first step. Once provincial and territorial alignment is in place, pan-Canadian coordination is possible. Limitations in recent health workforce modelling, including for the Caring for Canadians report^44^, could be substantially improved through the spread and scale of a combination of existing leading legal and regulatory practices.

## References

[1] World Health Organization . National Health Workforce Accounts: A Handbook. Geneva: World Health Organization; 2023.

[2] Simkin S Chamberland-Rowe C Damba C Sava N Lim T Bourgeault IL . Implementing leading practices in regional-level primary care workforce planning: lessons learned in Toronto. Healthc Manage Forum. 2023;36:15-20. 36239042 10.1177/08404704221117263 PMC9749563

[3] Bourgeault I Simkin S Chamberland-Rowe C . Poor health workforce planning is costly, risky and inequitable. CMAJ (Can Med Assoc J). 2019;191(42):E1147-E1148. 31636162 10.1503/cmaj.191241 PMC6805167

[4] Bourgeault I . A path to improved health workforce planning, policy & management in Canada: the critical coordinating and convening roles for the federal government to play in addressing 8% of its GDP. Sch Public Policy Publ. 2021;14(1): p. 7.

[5] Leslie K Demers C Steinecke R Bourgeault IL . Pan-Canadian registration and licensure of health professionals: a path forward emerging from a best brains exchange policy dialogue. Healthc Policy. 2022;18(1):17-25. 36103233 10.12927/hcpol.2022.26909 PMC9467269

[6] Sarabdeen J Chikhaoui E Ishak MMM . Creating standards for Canadian health data protection during health emergency–an analysis of privacy regulations and laws. Heliyon. 2022;8(5): p. 4. 10.1016/j.heliyon.2022.e09458 PMC9142849 35637667

[7] Saskatchewan Ministry of Health . Public representatives on self-regulating health profession councils. 2020. https://www.ehealthsask.ca/forms/Forms/Orientation-Manual-for-Public-Representatives-2020-Updated-May-2022_%281%29.pdf

[8] Regulated Health Professions Act. 1991, c 18. https://canlii.ca/t/56ds0

[9] Health Profession Regulators of Ontario . Quick facts about HPRO members. https://regulatedhealthprofessions.on.ca/

[10] Health Professions Act. 2000, c H-7. https://canlii.ca/t/56jck

[11] Disclosure of Information Regulation. Alta Reg 231/2013. https://canlii.ca/t/54wk2

[12] Health Professions and Occupations Act. Bill-36, 2022. https://www.bclaws.gov.bc.ca/civix/document/id/bills/billsprevious/3rd42nd:gov36-3

[13] Durcan R Richler E Steinecke R . Major regulatory reform comes to Canada. J Nurs Regul. 2023;14(2):43-48.

[14] The Regulated Health Professions Act. 2025, c 12. https://canlii.ca/t/56jqh

[15] First Session . Thirtieth Legislature of the Legislative Assembly of Saskatchewan Debates and Proceedings, 66. N.S; 2025. No. 18A. https://docs.legassembly.sk.ca/legdocs/Assembly/Debates/30L1S/20250401DebatesHTML.htm

[16] Health Professions Act, 2000, c H-7. https://canlii.ca/t/56kzt

[17] E-Health (Personal Health Information Access and Protection of Privacy) Act. 2008, c 38. https://canlii.ca/t/5571s

[18] Regulated Health Professions Network Act. 2012, c 48. https://nslegislature.ca/sites/default/files/legc/statutes/regulated_health_professions_network.pdf

[19] Scassa T . Public sector use of private sector personal data: towards best practices. Dalhous LJ. 2024;47:652.

[20] Bourgeault I Cohen D . Inclusive, integrated and enhanced data & digital infrastructure platforms for more timely and responsive health workforce planning and decision-making. Canadian Institutes of Health Research–funded project. 2024. Accessed January 26, 2026. https://cognit.ca/en/project/336402

[21] Personal Information Protection and Electronic Documents Act. 2000, c5. https://canlii.ca/t/56gxt

[22] Privacy Act. 1985, c P-21. https://canlii.ca/t/56jh1

[23] Personal Information Protection Act. 2003, c P-6.5. https://canlii.ca/t/56jlv

[24] Personal Information Protection Act. 2003, c 63. https://canlii.ca/t/566gk

[25] Gordon v Canada (Minister of Health) . Federal court (Canada). 2008. https://ca.vlex.com/vid/gordon-v-can-680872857

[26] Chiu P Louie-Poon S Leslie K Kung JY . Exploring the literature on racism and health practitioner regulation: a scoping review protocol. BMJ Open. 2024;14(7):e084084. 10.1136/bmjopen-2024-084084 PMC11253727 39002962

[27] Anti-Racism Data Act. 2022, c 18. https://canlii.ca/t/55g09

[28] Anti-Racism Act. 2024, c22. https://canlii.ca/t/56971

[29] Freedom of Information and Protection of Privacy Act, 1996, c 165. https://canlii.ca/t/56hrl

[30] Anti-Racism Data Act. 2022, c 18. https://canlii.ca/t/55g09

[31] Anti-Racism Act. 2017, c 15. https://canlii.ca/t/569mp

[32] Anti-Racism Directorate, Ontario . Data standards for the identification and monitoring of systemic racism. https://www.ontario.ca/document/data-standards-identification-and-monitoring-systemic-racism

[33] Chandrasekera U Choudhury S . Count us in: development and insights from Ontario’s equity and inclusion data initiative in social work and social service work regulation. Healthc Manag Forum. 2025;38:S35-S39. 10.1177/08404704251372073 41151077

[34] Augustine HJ . Employment match rates in the regulated professions: trends and policy implications. Can Public Policy. 2015;41(Supplement 1):S28-S47.

[35] Augustine HJ . Immigrant professionals and alternative routes to licensing: policy implications for regulators and government. Can Public Policy. 2015;41(Supplement 1):S14-S27.

[36] Sweetman A McDonald JT Hawthorne L . Occupational regulation and foreign qualification recognition: an overview. Can Public Policy. 2015;41(Supplement 1):S1-S13.

[37] Formoso A Curtis Maillet DG McDonald JT . From moose draws to health workforce planning: the uses of the provincial health insurance number in New Brunswick. Healthc Manag Forum. 2025;38:S65-S69. 10.1177/08404704251371572 41151078

[38] Ark TK Kesselring S Hills B McGrail K . Population data BC: supporting population data science in British Columbia. Int J Popul Data Sci. 2020;4(2):1133. 32935036 10.23889/ijpds.v4i2.1133 PMC7480325

[39] Dahl LT Katz A McGrail K , et al. The SPOR-Canadian data platform: a national initiative to facilitate data rich multi-jurisdictional research. Int J Popul Data Sci. 2020;5(1):1374. 34007883 10.23889/ijpds.v5i1.1374 PMC8104066

[40] Medical Council of Canada . National registry of physicians. https://mcc.ca/credentials-and-services/national-registry-of-physicians/

[41] College of Nurses of Ontario . Expanding nursys in Canada: advancing nationwide labour mobility. https://www.cno.org/news/expanding-nursys-in-canada?fbclid=IwQ0xDSwMHCxBjbGNrAwcKqGV4dG4DYWVtAjExAAEec14WU1cRPbLXp6eaSiG6atlVDBJDvRVYnNjRD5bCxPpJkT5-BVbmRNSGLRk_aem_ln48U5HknCyfhSWqARhGjw

[42] World Health Organisation . Health and Care Workforce, Global Strategy on Human Resources for Health: Workforce 2030, Report by the Director General. EB156/15. https://apps.who.int/gb/ebwha/pdf_files/EB156/B156_15-en.pdf

[43] Zagrodney K Bourcier D Gupta N , et al. Co-Developing an inclusive interprofessional health workforce minimum data standard for enhanced planning and decision-making: a Canadian case with international relevance. Health Policy. 2025;163:105485. 41275617 10.1016/j.healthpol.2025.105485

[44] Health Canada, Government of Canada . Caring for Canadians: Canada’s Future Health Workforce – the Canadian Health Workforce Education, Training and Distribution Study; 2025. https://www.canada.ca/en/health-canada/services/health-care-system/health-human-resources/workforce-education-training-distribution-study.html#a7

